# Expression of PAPP-A2 and IGF Pathway-Related Proteins in the Hip Joint of Normal Rat and Those with Developmental Dysplasia of the Hip

**DOI:** 10.1155/2019/7691531

**Published:** 2019-02-20

**Authors:** Yufan Chen, Haixiang Lv, Lianyong Li, Enbo Wang, Lijun Zhang, Qun Zhao

**Affiliations:** Department of Pediatric Orthopedics, Shengjing Hospital of China Medical University, Shenyang City 110004, China

## Abstract

Developmental dysplasia of the hip (DDH) is one of the major causes of child disability and early osteoarthritis. Genetic factors play an important role, but which still remain unclear. Pregnancy-associated plasma protein-A2 (PAPP-A2), a special hydrolase of insulin-like growth factor binding protein-5 (IGFBP-5), has been confirmed to be associated with DDH by previous studies. The aim of this study was firstly, to investigate the expression of PAPP-A2 and insulin-like growth factor (IGF) pathway-related proteins in normal rat's hip joints; secondly, to compare the variations of those proteins between DDH model rats and normal ones. The DDH model was established by swaddling the rat's hind legs in hip adduction and extension position. The hip joints were collected for expression study of fetal rats, normal newborn rats, and DDH model rats. Positive expression of PAPP-A2 and IGF pathway-related proteins was observed in all the hip joints of growing-stage rats. Ultimately, IGF1 was downregulated; insulin-like growth factor 1 receptor (IGF1R) showed an opposite trend in DDH rats when compared with normal group. The PAPP-A2 and IGF pathway-associated proteins may also be involved in the development of the rat's hip joint, which bring the foundation for further revealing the pathogenic mechanism of DDH.

## 1. Introduction

Developmental dysplasia of the hip (DDH) is an important cause of childhood disability, which can predispose a child to early onset degenerative changes and painful arthritis [1]. DDH is characterized by a rough joint surface, cystic degeneration, and deformation of the femoral head in the severe stage [2]. It affects approximately 1 to 5 per 1,000 newborns, among which about eighty percent are females. Yet, the etiology of DDH is complex and still unclear. Factors contributing to DDH include breech presentation, female sex, firstborn status, and oligohydramnios [3]. In the meantime, DDH has a considerable genetic etiology. Gene association mapping studies have hitherto identified several susceptible genes of DDH [4]. For example, mutations of cartilage formation-related proteins are associated with DDH [2].

Insulin-like growth factors (IGFs) play an important role in regulating cellular growth and development. IGFs are essential to the proliferation, differentiation, and apoptosis of osteoblasts. Meanwhile, they also regulate bone mineral density and skeletal growth [5, 6]. Insulin-like growth factor-binding protein (IGFBP) proteolysis directly modulates the initial step in IGF receptor signaling [7]. In the IGFBP family, IGFBP-5 is the most abundant IGFBP stored in the bone, which can act as a growth factor to enhance bone formation [8]. Pregnancy-associated plasma protein-A2 (PAPP-A2) is an IGFBP protease leading to the increased bioavailability of IGF1 by specifically cleaving IGFBP-5 and dissociating IGF1 from IGFBP-containing complexes [9].

PAPP-A2, a novel metalloproteinase, is a homologue of pregnancy-associated plasma protein A (PAPP-A) and a widely applied serum marker of pathological pregnancies during the first trimester, which shares 45% of its sequence with PAPP-A; however, it has a different physiological function in the growth regulatory system [10].

Variations of PAPP-A2 are related to human-complicated pregnancy, syndrome of progressive growth failure, reductions of cranial cartilage in zebrafish embryos, postnatal growth retardation in mice, and birthing in cattle [11–15]. In preeclamptic patients, maternal serum concentration of PAPP-A2 is increased which results from placental hypoxic exposure in utero [16]. Growth failure is commonly observed in patients with PAPP-A2 deficiency, which is characterized by thin and long bones, small chin, delayed dental eruption, and low bone mineral density (BMD) at the lumbar spine [12]. Treatment with recombinant human insulin-like growth factor-1 (rhIGF-1) is effective in promoting short-term growth in PAPP-A2 deficient patients and is also able to improve whole body BMD, bone mineral content (BMC), and lumbar spine BMD towards the normal range and results in an acceptable annual BMD increase [17, 18]. In zebrafish [14] *papp-a2* knockdown embryos, Alcian blue staining revealed a severe decrease or absence of Meckel's cartilage and cranial cartilages. Furthermore, Christians et al. [19] found that *Papp-a2* was responsible for the effect of a quantitative trait locus (QTL) affecting adult mice; postnatal growth retardation is obtained in *Papp-a2* knockout mouse demonstrates. Compared with the wild-type, *Papp-a2* knockout mice gain lower body weight; deletion of *Papp-a2* fails to the lengthening of some bones and affects pelvic girdle shape [14, 20]. Similar functional variation exists in cattle; Wickramasinghe and colleagues [15] identified and validated three single nucleotide polymorphism (SNP) which were associated with daughter calving ease, a measure of the intensity of dystocia, located in *PAPPA2* gene. Our previous case-control study with SNP analysis indicated a significant association between *PAPPA2* and DDH in the Han Chinese population [21].

Hence, we assume that due to the structural variation or decreased expression of the *PAPPA2* gene, the functional PAPP-A2 protein is reduced, which in turn reduces the degradation of IGFBP-5, and decreases the bioavailability of IGF1. PAPP-A2 may play a role in cartilage formation and cause the corresponding pathological changes of DDH through IGF signaling pathway. The main goals of our study are to confirm the expression of PAPP-A2, IGFBP-5, insulin-like growth factor 1 receptor (IGF1R), and IGF1 in the rat hip during different developmental stages and to examine the different expressions in the hips of normal rats and DDH model rats.

## 2. Materials and Methods

### 2.1. Animals

Wistar rats, 4-week-old, weighing 250-300 g, were obtained from the Animal Experimental Center, Shengjing Hospital of China Medical University. All the animals were housed in an environment with a temperature of 22 ± 1°C, relative humidity of 50 ± 1%, and a light/dark cycle of 12/12 hr. All animal studies (including the mouse euthanasia procedure) were in accordance with the regulations and guidelines of Shengjing Hospital of China Medical University Ethics Committee and conducted according to the AAALAC and the IACUC guidelines (protocol 2012PS13K).

## 3. Rats and Sample Collection

Sample collection was divided into two steps (Table 1):

**Step 1 .**

Five 21-day pregnant Wistar rats were sacrificed by intraperitoneal injection using 5% chloral hydrate (4 ml/kg). Consequently, their uterus were exposed, and their fetal rat hip joints were collected; sex selection was not performed among all these fetuses (1 day before birth, P21, *n* = 100).

Additional 71 newborn Wistar rats without sex selection were sacrificed 7 days (B7, *n* = 35) and 14 days (B14, *n* = 36) after birth, respectively. The rat's hip joints were collected in a similar way.

**Step 2 .**

A total of 113 newborn Wistar rats without sex selection were divided into control group (*n* = 60) and experimental group (*n* = 53). The control group was not exposed to any condition, while the experimental group was treated according to our previously reported method [22]. The DDH model was induced by modifying the swaddling position with fixation of the hind limbs in extension and adduction in newborn Wistar rats for 10 days. Half of the control group and the experimental group were sacrificed 7 days after birth (B7), while the rest were sacrificed 7 days later (B14). For the experimental group, the dislocation of the rat hip joint was confirmed by anatomical observation. All the hip joints were collected.

One-third of the samples (collected from Steps 1 and 2) were quickly rinsed in 0.01 M PBS and then immediately fixed in 4% paraformaldehyde phosphate buffer for 2 days. Samples were subsequently decalcified in 10% ethylenediaminetetraacetic acid (EDTA) for 15 to 20 days, dehydrated, and embedded in paraffin. Two-thirds of the samples were placed in a centrifuge tube frozen at -80°C for protein detection and RNA isolation.

### 3.1. Paraffin Sections and Immunohistochemistry

A total of 41 rats were used for immunohistochemistry in Step 1, and a total of 38 rats were used for immunohistochemistry in Step 2 (Table 1). Additionally, 20 slices for each acetabulum were analyzed among each individual rat. The paraffin hip specimens were sectioned (4 *μ*m thickness), deparaffinized in xylene, and rehydrated using descending grades of ethanol. Samples were then washed in PBS for 15 min, treated with 0.01 M citrate buffer (with pH 6.0), and then heated for 10 min on a microwave oven at 98°C. Consequently, sections were washed in PBS for 15 min, and then incubated with peroxidase blockers for 30 min at room temperature. After washing in PBS for an additional 15 min, samples were incubated with normal nonimmunized animal serum at room temperature for 30 min and then incubated with one of the following antibodies: polyclonal rabbit anti-rat PAPP-A2 antibody (1 : 40 dilution, Lifespan, USA), polyclonal rabbit anti-human IGFBP-5 (1 : 200 dilution, Abcam, UK), polyclonal rabbit anti-rat IGF1R (1 : 50 dilution, Sigma-Aldrich, Germany), and monoclonal antibody rabbit anti-human IGF1 (1 : 100 dilution, Lifespan, USA) at 4°C overnight; PBS was used as a negative control. Biotinylated secondary antibodies were applied to samples for 20 min followed by streptavidin-HRP incubation (40 min) at room temperature. Sections were visualized with DAB (Maixin Biotechnology Development Co. Ltd., China) under the light microscope (Nikon E800, Japan). Samples were counterstained using haematoxylin (Maixin Biotechnology Development Co. Ltd., China) diluted 1 : 5 in sterile water, and the cell cytoplasm was brownish yellow when the results were positive. The sections were observed by 40 times and 400 times visual field, to determine the main distribution of positive cells and the general trend of change. The expressions of PAPP-A2, IGFBP-5, IGF1R, and IGF1 in the germinal cell layer, proliferative cell layer, and hypertrophic cell layer of the epiphyseal cartilage located both in acetabulum and femoral head were observed and analyzed by NIS-Elements Basic Research (ver. 2.1, Nikon, Japan).

### 3.2. Protein Extraction and Western Blot

A total of 50 mg of rat acetabular tissue were homogenized in 500 *μ*l of RIPA lysis buffer (P0013B, Biyuntian Biotechnology Co. Ltd., China) and PMSF (ST506, Biyuntian Biotechnology Co. Ltd., China). Samples were then incubated on ice for 30 min and then centrifuged at 12,000 rpm at 4°C for 15 min. The supernatant was then collected, and the protein levels were determined using the modified Lowry method. Proteins were mixed with SDS ×6 loading buffer (TransGen Biotech Co. Ltd., China) boiled for 5 min and run through a 4% stacking and 10% separating polyacrylamide gel (P0012A, Biyuntian Biotechnology Co. Ltd., China). The gel was equilibrated in a transfer buffer and transferred onto a PVDF membranes, which were activated by methanol. Membranes were blocked for an hour and a half at room temperature in 5% skimmed milk powder (BD, USA) and then incubated in a solution containing the primary antibody against PAPP-A2 (1 : 200 dilution), IGFBP-5 (1 : 1000 dilution), IGF1R (1 : 500 dilution), and IGF1 (1 : 1000 dilution) diluted with the titer of the corresponding antibody using 1% BSA antibody buffer at 4°C overnight. Consequently, the membranes were washed 3 times with TBST (0.24% Tris, 0.8% sodium chloride, and 0.1% Tween 20) and incubated in a solution containing the secondary antibody which was diluted with the corresponding antibody titer using 1% BSA antibody buffer for 2 hours. The membranes were repeatedly washed 3 times with TBST. Finally, the membranes were covered with ECL fluid (NCI5079, Thermo Fisher Scientific, USA) for 2 min and observed in an automatic electrophoresis gel imaging analyzer (4/1246, SYOR, USA).

### 3.3. RNA Isolation and Real-Time PCR

Rat acetabular samples were taken out of the -80°C refrigerator and homogenized with pestles after they were frozen in liquid nitrogen. Each tissue homogenate (100 mg) was added to 1 ml TRIzol reagent (Invitrogen, USA) at room temperature and then centrifuged with high-speed centrifuge (Eppendorf, Germany) at 12,000 rpm at 4°C for 15 min. Samples were then mixed with 0.2 ml chloroform per 1 ml TRIzol for 45 sec, incubated at room temperature for 10 min, and centrifuged at 12,000 rpm at 4°C for 15 min. Consequently, the supernatant was removed, and 0.4 ml isopropanol was added to the aqueous phase. Samples were incubated at room temperature for 10 min and then centrifuged at 12,000 rpm at 4°C for 15 min. The RNA precipitate was washed twice with 70% and 100% ethanol, air dried, and resuspended in 30 *μ*l molecular grade water. Quantification and analysis of isolated RNA were performed using the NanoVue spectrophotometer (GE Healthcare Bio-Sciences AB, Germany). cDNA was reverse transcribed from RNA by PrimeScript™ RT Reagent Kit (RR047A, Takara Bio, Japan), and real-time PCR was performed to measure the levels of *Pappa2* mRNA, *Igfbp5* mRNA, *Igf1* mRNA, and *Igf1R* mRNA. *Gapdh* was used as loading control. Primer sequences are shown in Table 2. PCR was carried out using the LightCycler detection system (D-68298, Roche Molecular Biochemical, Germany) with samples tested in duplicate on 96-well plates. Amplicons were generated in a two-step PCR (95°C for 30 sec for predegeneration, 95°C for 5 sec, and 60°C for 30 sec for 40 cycles) followed by melting curve analysis to exclude contamination of nonspecific products. Each sample was amplified for 40 cycles, and the cycle at which the signal rose above a fixed threshold (Ct) was determined, and relative quantity (RQ) was calculated with Ct mean.

### 3.4. Statistical Analysis

All data were expressed as mean ± standard deviation. SPSS statistical software (17.0, IBM, USA) was used to analyze the experimental data by single factor analysis of variance; LSD was used for posthoc test and independent sample *t* test. *P* < 0.05 was considered statistically significant.

## 4. Results

### 4.1. Expression and Localization of PAPP-A2 and IGF Pathway-Associated Proteins in the Normal Rat Hip Joints at Different Developmental Stages

**Figure 1:**
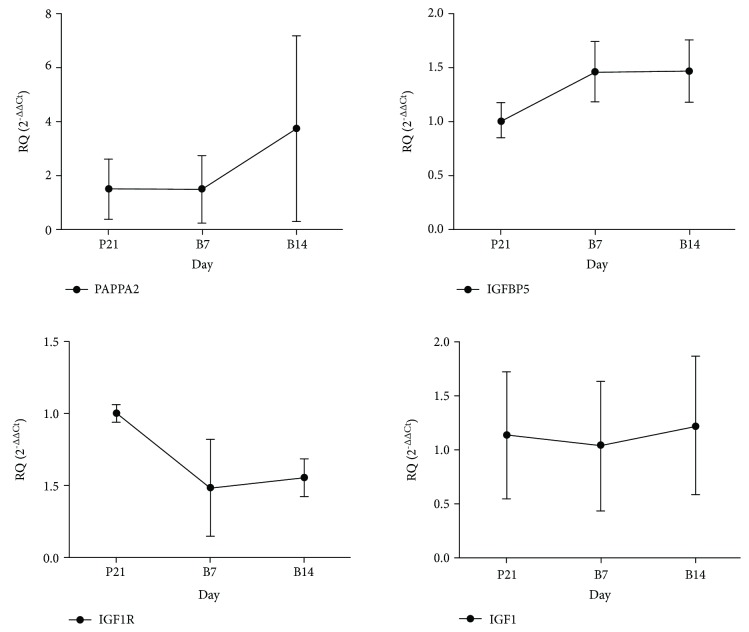
mRNA expression levels of *Pappa2* and *Igf* pathway-associated gene in rat hip joints during different growth stages. mRNA expression levels of *Pappa2*, *Igfbp5*, *Igf1R* (*P* = 0.049), and *Igf1*. P21 (*n* = 40), one day before birth; B7 (*n* = 11), seven days after birth; and B14 (*n* = 12), fourteen days after birth. ^∗^*P* < 0.05.

Quantitative RT-PCR analysis was used to verify the expression levels of *Pappa2*, *Igfbp5*, *Igf1R*, and *Igf1* on different-aged rat hip joints. *Gapdh* was used as a standardizing sample, since its mRNA levels did not change between different time phases.*Pappa2*, *Igf1*, and *Igf1R* transcript levels showed the same trends. At B7, no significant changes in *Pappa2* and *Igf1* were observed, while *Igf1R* expression (*P* = 0.049) was significantly reduced compared with other time phases (Figure 1).

**Figure 2:**
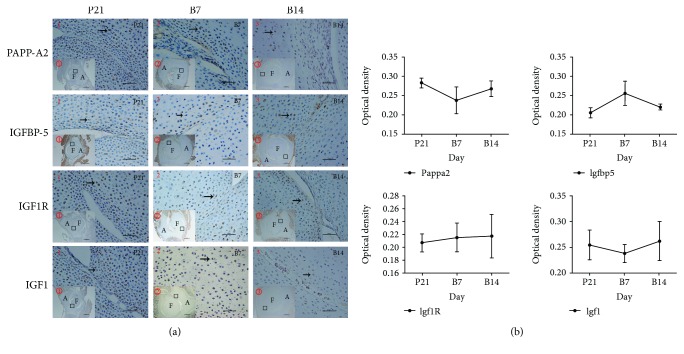
Immunohistochemistry results of PAPP-A2, IGFBP-5, IGF1R, and IGF1 in rat hip joints during different growth stage. P21 (*n* = 17), B7 (*n* = 12), and B14 (*n* = 12) represent day 1 before birth, day 7, and day 14 after birth, respectively. (a) (1, 2, and 3 original magnifications ×400; ①, ②, and ③ original magnification ×40). The black arrow indicates positive cells. A: acetabulum; F: femoral head. (b) PAPP-A2, IGFBP-5, IGF1R, and IGF1 in normal hip joints with time varying.

Immunohistochemistry was used to investigate the localization of PAPP-A2, IGFBP-5, IGF1R, and IGF1 in the germinal cell layer, proliferative cell layer, and hypertrophic cell layer of the epiphyseal cartilage of acetabulum and femoral head. Positive expressions of these proteins were found in the hip joints at P21, B7, and B14; proteins were distributed in the epiphyseal cartilage of acetabulum and femoral head (Figure 2(a)). No significant difference was noted between different time phases, but the PAPP-A2 and IGF1 had the lowest expression, while IGFBP-5 had the highest expression at B7. Furthermore, no significant difference of IGF1R expression was found during different time phases (Figure 2(b)).

**Figure 3:**
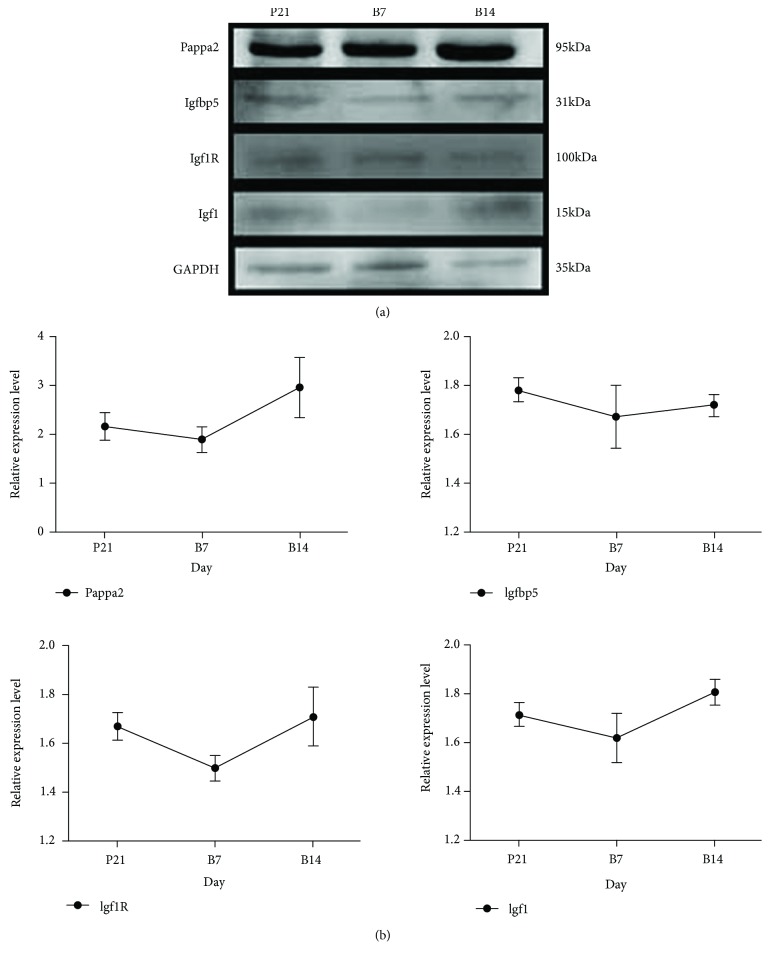
Protein expression of PAPP-A2, IGFBP-5, IGF1R, and IGF1 in rat hip joints during different growth stages measured by Western blot. P21 (*n* = 43), B7 (*n* = 12), and B14 (*n* = 12) represent day 1 before birth, day 7, and day 14 after birth, respectively. ^∗^*P* < 0.05.

Moreover, Western blot data suggested a similar expression of PAPP-A2, IGF1, and IGF1R protein in the rat's hip joints. Significant reductions in PAPP-A2 (*P* = 0.048), IGF1 (*P* = 0.049), and IGF1R (*P* = 0.040) were found at B7 compared with those in P21 and B14, whereas no changes were observed in the expression of IGFBP-5 (Figure 3).

### 4.2. Establishment of a DDH Rat Model

**Figure 4:**
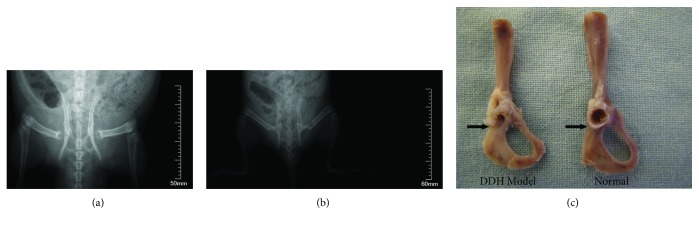
Anteroposterior pelvic radiographs and acetabular tissue of DDH model hip and normal hip. (a) Dislocation in both right and left hip of DDH model. (b) Anteroposterior pelvic radiographs of normal hip. (c) A dysplastic true acetabulum of DDH model hip (left) and normal acetabulum of normal hip (right).

Rats were swaddled for the first ten days after birth to build a DDH model. The DDH model was induced by modifying the swaddling position with fixation of the hind limbs in extension and adduction in newborn Wistar rats for 10 days. The anteroposterior pelvic region and acetabular tissue of the DDH model's hip were then examined using radiography (Figure 4).

### 4.3. Expression and Localization of PAPP-A2 and IGF Pathway-Associated Proteins in the Control Group and Experimental Group

**Figure 5:**
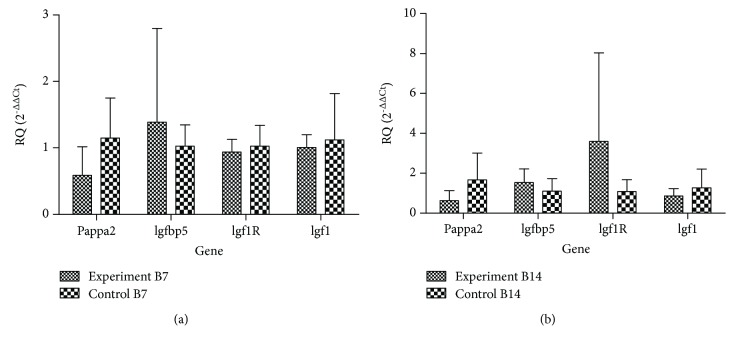
mRNA expression levels of *Pappa2* and *Igf* pathway-associated gene in the control group and experimental group. (a) The expression of *Pappa2*, *Igfbp5*, *Igf1R*, and *Igf1* at B7 (control *n* = 10 and experimental *n* = 8). (b) The expression of *Pappa2*, *Igfbp5*, *Igf1R,* and *Igf1* at B14 (control *n* = 10 and experimental *n* = 9).

RT-PCR was used to detect the expression of these genes. Seven days after birth, *Pappa2*, *Igf1*, and *Igf1R* were downregulated, and *Igfbp5* was upregulated in the experimental group. Nevertheless, at 14 days after birth, *Pappa2* and *Igf1* were downregulated, while *Igfbp5* and *Igf1R* were upregulated compared to the control group. However, these results were nonsignificant at both B7 and B14 (Figures 5(a) and 5(b)).

**Figure 6:**
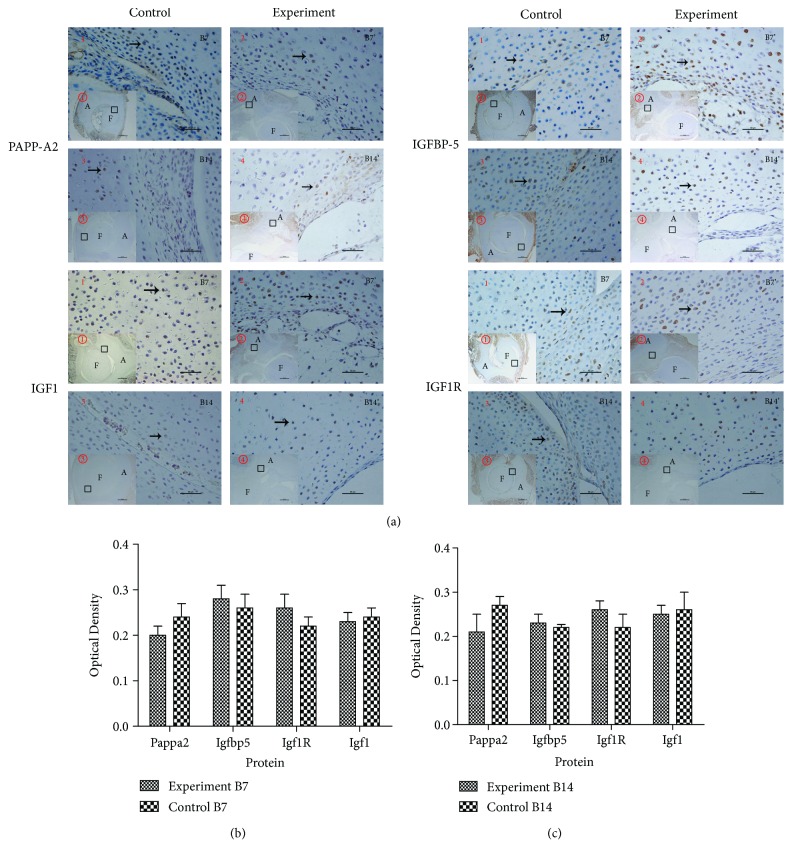
Localization of PAPP-A2 and IGF pathway-associated proteins in the control group and experimental group. (a) (1, 2, 3, and 4 original magnification ×400; ①, ②, ③, and ④ original magnification ×40). The black arrow indicates positive cells. A: acetabulum; F: femoral head. B7 and B14 represent day 7 and day 14 after birth, respectively. (b) Results of PAPP-A2, IGFBP-5, IGF1R, and IGF1 at B7 (control *n* = 10 and experimental *n* = 9). (c) Results of PAPP-A2, IGFBP-5, IGF1R, and IGF1 at B14 (control *n* = 10, experimental *n* = 9).

Immunohistochemistry was used to further investigate the localization of PAPP-A2, IGFBP-5, IGF1R, and IGF1 in the germinal cell layer, proliferative cell layer, and hypertrophic cell layer in the epiphyseal cartilage of acetabulum and femoral head of the control group and the experimental group. Positive expressions of these proteins were observed at B7 and B14; proteins were distributed in the acetabular cartilage and femoral head cartilage (Figure 6(a)). Compared to the control group, no significant difference was found in the experimental group (Figures 6(b) and 6(c)).

**Figure 7:**
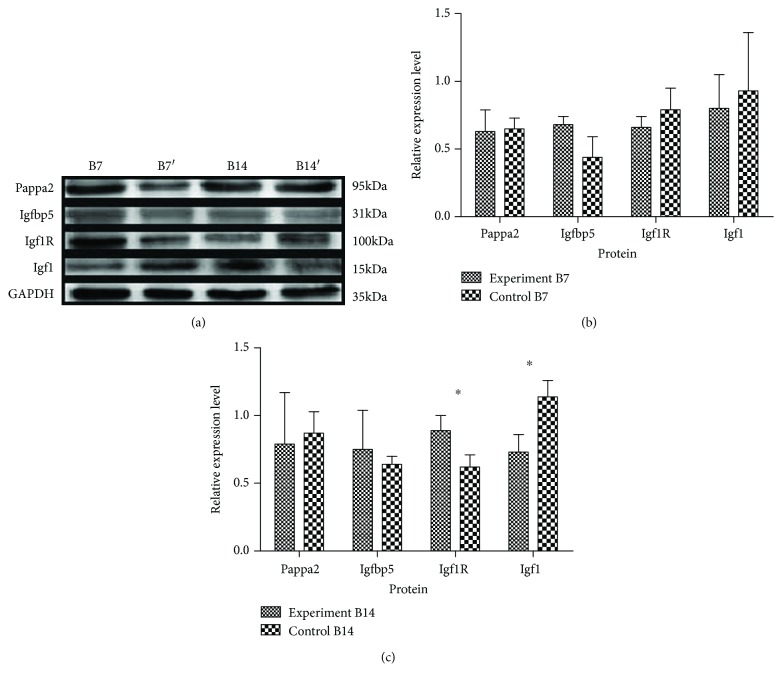
Western blot analyses of PAPP-A2 and IGF pathway-associated proteins in the control group and experimental group. (a) Electrophoretic bands of PAPP-A2 and IGF pathway-associated proteins. B7 and B14, control group 7 and 14 days after birth; B7' and B14', experimental group 7 and 14 days after birth, respectively. (b) Statistical analysis of PAPP-A2, IGFBP-5, IGF1R, and IGF1 protein expression at B7 (control *n* = 10, experimental *n* = 9). (c) Statistical analysis of PAPP-A2, IGFBP-5, IGF1R (*P* = 0.030), and IGF1 (*P* = 0.017) protein expression at B14 (control *n* = 10, experimental *n* = 9). ^∗^*P* < 0.05.

The effect of swaddling was further examined by Western blot (Figure 7(a)), at B7, lower PAPP-A2, IGF1R, and IGF1 expression and higher IGFBP-5 expression were detected in the experimental group compared to the control group; however, no significant difference was noted between the groups. Nevertheless, at B14, the expression of IGF1 was significantly downregulated in the rat hip joint of the experimental group compared with the control group hip, but the expression of IGF1R indicated an opposite trend (Figures 7(b) and 7(c)). There was no animal who died before the end of the swaddling.

## 5. Discussion

In the present study, we investigated the expression of PAPP-A2, IGFBP-5, IGF1R, and IGF1 in the hip joints of normal rats and DDH model rats. Additionally, three different time phases (P21, B7, and B14) were chosen to examine those variations within the growth and development in normal rats.

IGF1 is a principal growth-promoting signal for skeletal development. IGF1R acts as a receptor for IGF1 to promote the proliferation and differentiation of chondrocytes in growth plate by inhibiting the PTHrP pathway [23]. Previous study showed a rational mechanism in osteoblast-specific PAPP-A2 deletion mouse where local IGFBP-5 level increase led to a reduction of IGF availability [24]. Moreover, during chondrocytes development, IGF1 was upregulated while IGFBP-5 was downregulated [25]. In our study, PAPP-A2 and the IGF signaling pathway-related proteins were both expressed in the femoral head and acetabular cartilage of the hip joints. In addition, PAPP-A2, IGF1, and IGF1R showed the similar trends at different time phases. Accordingly, we can assume that PAPP-A2, IGFBP-5, and IGF signaling pathway-related proteins are positively expressed in normal rat hip joints. During developmental stage, the expression levels of these proteins remained constant and did not change with time after birth. In normal rats, the Western blot data showed a different trend in IGFBP-5, but RT-PCR results implied that there might be other signaling pathways involved in the development of the femoral head and acetabular cartilage. Since we only chose three different time phases for the observation of the cartilaginous development, it was devoid of reliability to make a strong conclusion.

*Pappa2* knockout mouse pelvic girdles obviously reflect variation in the shape of the ischium, the length of the pubis, and to a lesser extent, the width of the end of the ilium. There is also substantial variation in sexual dimorphism [20]. Mee [26] suggested that *PAPPA2* might regulate the maternal pelvic size. Clinical studies showed that the structural abnormalities might exist in the pelvis of patients with DDH; additionally, abnormalities of the acetabulum are not only caused by local dysplasia around the hip but also ascribed to the morphologic development of the entire pelvis [27]. In our previous study [21], we performed a genome-wide scan with Affymetrix 10K SNP arrays on four generations of Chinese family, which included 19 healthy members and five patients with DDH. The results indicated that SNP locus rs726252, which was linked to the family, is in *PAPPA2* gene, further suggests an association of *PAPPA2* with sporadic DDH susceptibility in the Chinese Han population. Dauber and colleagues [28] found two different homozygous mutations in *PAPPA2* associated with a novel syndrome of growth failure, which was characterized by progressive growth failure, moderate microcephaly, and thin long bones, indicating that PAPP-A2 was a key regulator of human growth and IGF1 bioavailability by modulating the proportion of IGF1 that was free or bound to its IGFBP. IGF1, IGFBP-5, and IGF1R were essential for the regulation of cartilage development in the IGF signaling pathway, and their balance can affect the development of cartilage [29–31]. At the same time, IGF signaling pathway can also promote human fibroblasts to synthesize type I collagen and to maintain stable collagen content in connective tissues [32]. IGF is closely related to the development of cartilage and collagen synthesis of fibroblasts, and these two aspects are precise pathological factors that occur in DDH. We hypothesized that abnormal expression level of *PAPPA2* may promote pelvic morphologic development associated with DDH. We established a DDH rat model and found that IGF1 was downregulated, IGF1R showed an opposite trend, while other proteins remained unchanged in 14-day-old model. The expression changes of IGF1 and IGF1R were observed in the DDH model caused by the straight-leg swaddling; the PAPP-A2 and IGF pathway-associated proteins may also be involved in the development of the rat hip joint. However, this study could not confirm whether the abnormal expressions of these proteins were primary or secondary changes in the hip joints caused by environmental factors (abnormal biological forces). At the same time, there was no analysis on the severity of DDH model pathology, and therefore, the correlation between the severity of DDH and IGF pathway-associated proteins could not be clearly defined.

## 6. Conclusion

We confirmed that PAPP-A2 and IGF pathway-associated proteins were positively expressed in the rat hips at the developmental stage, indicating that they were involved in the hip development. At the same time, these key proteins were lowly expressed in the hip of the DDH model, indicating that the abnormalities of IGF pathway-associated proteins may be related to the development of DDH. However, no changes in PAPP-A2 mRNA or protein levels were observed in animals with DDH caused by environmental factors. Therefore, we are unable to establish a relation between PAPP-A2 and DDH model. Further studies are necessary to reveal the mechanisms at the cellular level.

## Figures and Tables

**Table 1:** Animal distribution of each group.

	Step 1			Step 2			
	P21	B7	B14	CB7^∗^	EB7^∗^	CB14^∗^	EB14^∗^
PCR	40	11	12	10	8	10	9
Western blot	43	12	12	10	9	10	9
Immunohistochemistry	17	12	12	10	9	10	9

Note: ^∗^ CB7 means B7 in control group; EB7 means B7 in experimental group; CB14 means B14 in control group; EB14 means B14 in experimental group.

**Table 2:** Primer sequences used in qRT-PCR.

Gene	Forward primer (5′ → 3′)	Reverse primer (5′ → 3′)
*Pappa2*	CAAGACCTGCTTTGACCCTGA	GCACTGAGCTGGCAAAGTAGATG
*Igfbp5*	CTACGGCGAGCAAACCAAGATA	GGCCTTCAGCTCGGAAATG
*Igf1R*	CGGGATCTCATCAGTTTCACAGTC	TCCTTGTTCGGAGGCAGGTC
*Igf1*	GCACTCTGCTTGCTCACCTTT	TCCGAATGCTGGAGCCATA
*Gapdh*	GCTGGTCATCAACGGGAAA	CGCCAGTAGACTCCACGACAT

## Data Availability

The data used to support the findings of this study are available from the corresponding author upon request.
